# Palmar Ulnar Artery Perforator Free Flap for Fingertip Reconstruction: Anatomical and Clinical Study

**DOI:** 10.1155/2018/2862879

**Published:** 2018-05-09

**Authors:** Chang Sik Pak, Ji-In Jeon, Yujin Myung, Yung Ki Lee, Byung Jun Kim, Jae Hoon Jeong, Baek-Kyu Kim

**Affiliations:** ^1^Department of Plastic and Reconstructive Surgery, Seoul National University Bundang Hospital, Seongnam, Republic of Korea; ^2^Department of Plastic and Reconstructive Surgery, Kyung Hee University Hospital, Seoul, Republic of Korea; ^3^Department of Plastic and Reconstructive Surgery, Seoul National University Hospital, Seoul, Republic of Korea

## Abstract

**Background:**

Fingertips are a common site for hand injuries. The ideal substitute for fingertip pulp is tissue that matches texturally with minimal donor site morbidity. We described anatomical findings from cadaveric studies and the reliability of the palmar ulnar artery perforator (PUAP) free flap techniques for the reconstruction of fingertips injuries.

**Methods:**

The cadaveric study involving 8 hands was conducted to illuminate the anatomy of the hypothenar region. We investigated the emerging point of perforators, diameter of the artery at the origin, and the length of the pedicle. Forty-four patients with fingertip injuries underwent fingertip reconstruction using PUAP free flaps. Data on the baseline patient demographics, hospital courses, and flap sizes were obtained retrospectively. The 6-month postoperative sensory capacity was determined using a 2-point discrimination test.

**Results:**

The cadaveric study found that the PUAP pedicles arose from the superficial palmar arch, the mean length of pedicles, and the diameter and location of perforators were also analyzed. The PUAP flap sizes varied from 2.0 × 2.3 cm to 2.5 × 3.5 cm, and the mean operative time was 124 min. In all cases, we performed neurorrhaphy for fingertip sensory restoration. All donor sites were closed primarily, and the 2-point discrimination test result was 5.7 ± 0.87 mm 6 months after surgery.

**Conclusions:**

We confirmed the anatomical consistency of the PUAP. Among the numerous reconstruction options available for fingertip injuries, the PUAP free flap is a useful fingertip reconstruction option.

## 1. Introduction

Fingertip injuries are common and challenging problems for hand surgeons. Furthermore, fingertip amputations can affect patients physically and psychologically. Among Asian cultures, people prefer to preserve the body parts as they received them from their parents, and they favor fingertip reconstruction over amputation to show respect to their parents. Although replantation surgery is the best treatment for fingertip amputation, this option may be impossible due to several reasons, such as loss of distal part of digit. Numerous fingertip reconstruction techniques have been introduced that range from simple sutures to free tissue transfer. With advances in, and the worldwide use of, microsurgical techniques, free tissue transfer is no longer a difficult surgical procedure as reconstructive option for fingertip amputation and there are many free tissue transfer methods being developed and free toe pulp transfer is widely used [1].

Recently, the use of cutaneous perforators has been investigated in fingertip reconstruction, which represents a quantum leap forward in microreconstructive surgery for this area of the body. For example, a perforator free flap has been used successfully with a single safe cutaneous perforator. The arterial perforator flap offers several advantages, including superior accuracy, its small size, and a design that creates a thin flap, which ensures that donor site morbidity is minimized. The flap fulfilled the qualities which were (1) the ability to restore the finger pulp sufficiently while controlling flap thickness; (2) successful sensory recovery; (3) an excellent pedicle size match; and (4) anatomical consistency with the donor site. In this study, we used the palmar ulnar artery perforator (PUAP) free flap technique that was devised during an anatomical study by Hwang et al. [2]. We described anatomical findings from a cadaveric studies and the reliability of the PUAP free flap techniques for the reconstruction of fingertips injuries.

## 2. Methods

### 2.1. Cadaveric Study

**Figure 1:**
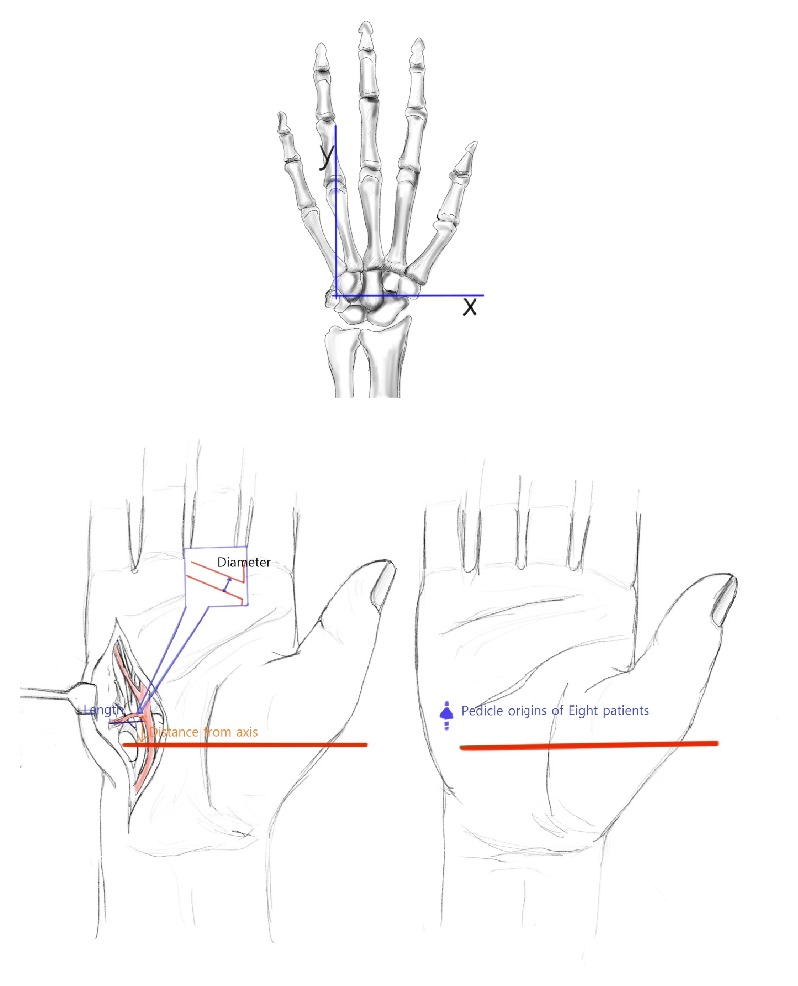
Palmar ulnar artery perforator (PAUP) arising location based on *x*, *y* coordinates on the hand. We evaluated the following parameters: the distance from the line between the pisiform and hamate bones set up as *x*-axis to the point at which the perforators emerge; the diameter of the artery at the origin; and the length of the pedicle from the origin to the bifurcation.

Anatomical consistency of the donor site is essential for flap reconstruction. Hence, we conducted a cadaveric study to establish the anatomy of the hypothenar region. The cadaveric study involved 8 amputated forearms from fresh preserved cadavers. Red latex was injected into the axillary arteries using the 18 Fr catheters. We evaluated the following parameters: (1) the distance from the line between the pisiform and hamate bones set up as *x*-axis to the point at which the perforators emerge; (2) the diameter of the artery at the origin; and (3) the length of the pedicle from the origin to the bifurcation (Figure 1).

### 2.2. Clinical Study

This study was approved by Seoul National University Bundang Hospital Institutional Review Board and Ethics Committee and followed the guidelines of the 1975 Declaration of Helsinki. From April 2011 to April 2016, 44 patients with fingertip injuries, comprising 10 women and 34 men, underwent reconstruction using PUAP free flaps. Patients were eligible for PUAP free flap reconstruction if they had a Zone I injury with bone exposure and they had not experienced distal amputation. The PUAP free flap was designed for all of the patients who required fingertip reconstruction. Data about the baseline patient demographics, hospital courses, and flap sizes were obtained retrospectively. The 6-month postoperative sensory capacity was measured using a 2-point discrimination test with a caliper whose two indicators varied in distance from 1 mm to 10 mm.

**Figure 2:**
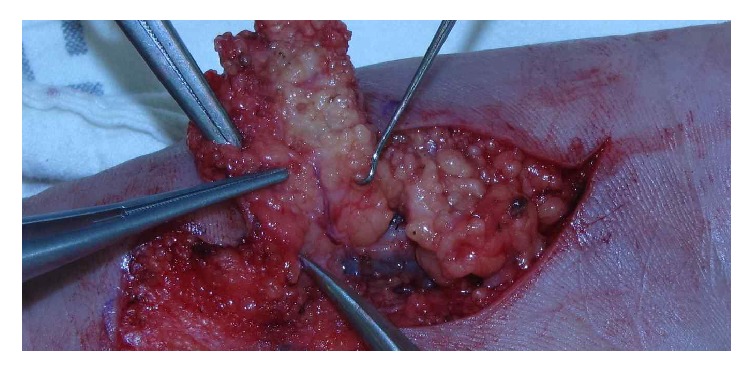
Intraoperative view of the palmar ulnar artery perforator (PAUP). The palmar ulnar artery perforator arising from the superficial palmar arch was identified.

*Operative Technique*. The operations were done either under general anesthesia or under local anesthesia. While we used a tourniquet in upper arm only for general anesthesia, we performed digital nerve blocks using 2% lidocaine and used finger tourniquet for local anesthesia. At this time, local anesthetic was injected directly without ulnar nerve blocks in donor site. First, the recipient finger was prepared by finding the recipient artery, vein, and nerve. To determine the size of the injury, the flap elevation of the hypothenar region was followed. The hypothenar ulnar artery perforator arises at the intersection between the transverse *x*-axes, which runs through the pisiform and hamate bones, and proximal to a distal *y*-axis, which runs through the pisiform bone and fourth interdigital web [2]. The flap was designed around the emerging points of perforators based on the previous anatomical study, and the cutaneous veins were marked using a gentian violet surgical marker pen. Under loupe magnification, a careful incision was made from the medial side. After a secure pedicle length was obtained, the venous channel was dissected with the expectation that the flap would be nourished by 2-3 cutaneous veins (Figure 2). The cutaneous superficial veins were identified in all cases and were preserved for use if the concomitant veins of the palmar ulnar artery were too small for the creation of anastomoses. Flap circulation was confirmed after the tourniquet was released. Once the flap was inset, the vascular anastomosis was created under a surgical microscope using 10-0 sutures. After the donor site was closed primarily, the postoperative flap was monitored, and medication was administered in accordance with the routine practice for flap surgery.

## 3. Results

### 3.1. Cadaveric Study

**Figure 3:**
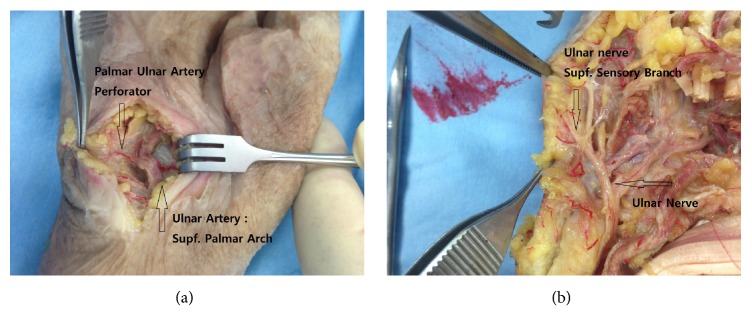
Anatomic study on cadavers. (a) The palmar ulnar artery perforator arises from the superficial palmar arch. The mean diameter of the perforator was 0.9 mm and the pedicle length was 11.25 mm. The perforator usually arises 20.0 mm distal from the *x*-axis, which is the line between the pisiform and hamate bones. (b) The superficial sensory branch of the ulnar nerve covered the hypothenar region.

The cadaveric study on two male and two female cadavers, a total of 8 hands, showed that the PUAP arose from the superficial palmar arch, the mean diameter at the origin of the perforator was 0.9 ± 0.15 mm, the mean pedicle length from the origin to the flap was 11.25 ± 1.67 mm, and the perforator usually arose 20.0 ± 2.88 mm distal from the *x*-axis, which is the line between the pisiform and hamate bones (Table 1). The superficial sensory branch of the ulnar nerve covered the hypothenar region (Figure 3).

### 3.2. Clinical Study

From April 2011 to April 2016, 44 patients, comprising 10 women and 34 men, underwent fingertip reconstruction using the PUAP free flap technique (Table 2). Their ages ranged from 20 to 62 years, and their mean age was 42.7 years. The fingertip injuries were caused by knives (*n* = 11), crushing (*n* = 33), and the failure of primary replantation surgical procedures at local orthopedic clinics (*n* = 7). The flap sizes varied from 2.0 × 2.3 cm to 2.5 × 3.5 cm, and the mean operative time was 124 minutes (98–220 minutes). In all cases, we performed neurorrhaphy to restore the fingertips' sensory function. We performed venous anastomoses in 43 cases, with the exception of 1 patient, and we used the superficial cutaneous vein in 22 cases and the vena comitans in 21 cases. The recipient site vein was severely crushed in 1 patient, we did not undergo venous anastomosis; however, complete healing was achieved, despite partial flap loss caused by venous congestion, and no further surgical interventions were required. All donor sites were closed primarily, and the mean 2-point discrimination test result was 5.7 mm 6 months after surgery. The tactile sense was normal considering that the general distance of 2-point discrimination test on fingertip is 2 to 8 mm [3].

**Figure 4:**
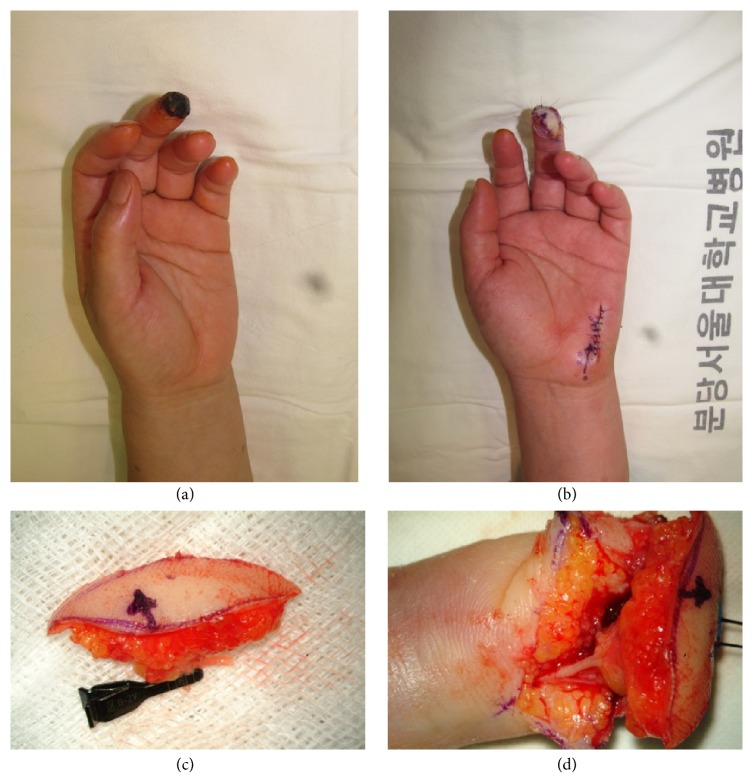
Case of patient. A 52-year-old male patient who had been crushed by a presser machine visited our outpatient clinic after the complete failure of the composite graft that he had received at a local clinic. The patient was seeking fingertip reconstruction following graft failure 16 days after his first operation. We removed the distal necrotic tissue under local anesthesia and elevated the PUAP flap, which was 2.0 × 3.0 cm and had sensory innervation and 2 venous channels. (a) Fingertip necrosis following replantation surgery. (b) Immediately after the operation. (c) After flap harvesting. (d) After the anastomosis of the artery.

**Figure 5:**
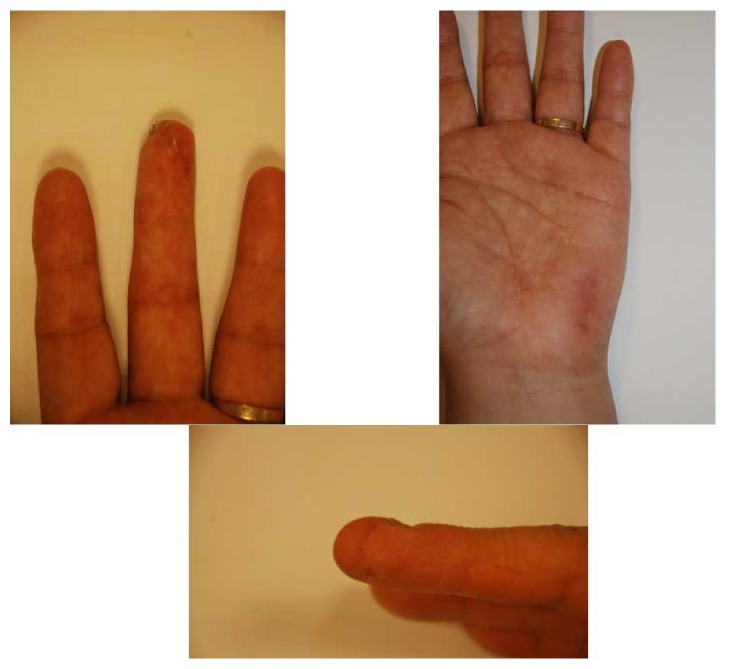
Photograph after 5 months after surgery. The patient underwent a successful reconstruction without any complication.

*Case*. A 52-year-old male patient who had been crushed by a presser machine visited our outpatient clinic after the complete failure of the composite graft that he had received at a local clinic. The patient was seeking fingertip reconstruction following graft failure 16 days after his first operation. We removed the distal necrotic tissue under local anesthesia and elevated the PUAP flap, which was 2.0 × 3.0 cm and had sensory innervation and 2 venous channels (Figure 4). The patient underwent a successful reconstruction without any complication (Figure 5).

## 4. Discussion

The hypothenar muscles and the transverse carpal ligament are covered by a layer of adipose tissue that is supplied by at least 3 arterial branches originating from the ulnar artery in Guyon's canal. Previous cadaveric studies have shown that the branches of the segmental ulnar artery arise approximately every centimeter, beginning at the distal wrist flexion crease, and that their diameters range from 0.7 to 1.5 mm [2]. The ulnar digital nerve of the small finger runs deep into the distal third of the fat pad after branching from the ulnar nerve in Guyon's canal [2]. The skin over the hypothenar region is nourished through the hypothenar muscle and fascia by myocutaneous or fasciocutaneous perforators.

The palmar artery perforator flap is based on multiple fasciocutaneous perforators that arise from the ulnar palmar digital artery of the little finger [4]. Several options should be considered before undertaking fingertip reconstructions following Zone I injuries, even in cases with modest deficits. Reconstruction using an advancement flap, cross-finger flap, and an innervated cross-finger pulp flap are very popular, and pedicled flap within hand matches the texture of the fingertips very closely, the loss of the main pedicle is a major drawback of this reconstruction technique. Advances have been made in microsurgical techniques, and they are used worldwide, such as in cases of free tissue transfers. Recently, the use of the cutaneous perforator has been investigated, because a cutaneous perforator free flap requires the use of only one safe perforator.

Free tissue transfer may be categorized as originating from the lower limb or the upper limb, depending on the donor site used for fingertip reconstruction. For example, Huang et al. reconstructed fingertips from lower limbs using medial plantar artery perforator free flaps, Koshima et al. used a partial toe transfer, and Gu et al. used a partial second toe pulp free flap [1, 5, 6]. The partial second toe transfer method is now widely used and offers excellent size and color matching. Using the upper limb as the donor site, Tsai et al. used a thenar minifree flap to revascularize a finger in 1991, while Inada et al. and Simsek et al. used free dorsoulnar perforator flaps for the reconstruction of digits [7–9]. In 2000, Omokawa et al. used a PUAP pedicled flap in finger reconstruction, and Hwang et al. demonstrated the use of the ulnar artery perforator in a cadaveric study [2, 4].

To demonstrate the anatomical consistency of the PUAP free flap, we performed an anatomical cadaveric study, which showed that the perforator usually arises at a mean distance of 20.0 mm from the *x*-axis, which is the line between the pisiform and hamate bones. When designing the flap, surgeons should consider the distance at which the perforator emerges. Furthermore, the arterial diameter was 0.9 mm when the actual diameter should be less than 0.9 mm. During cadaver preparation, the arterial diameter requires enlargement of the small perforator, which can be challenging for surgeons specializing in microsurgery and super microsurgery. The pedicle length from the origin was 11.25 mm, which was sufficient for the anastomosis of the vessel in Zone I, without the requirement for an additional vein graft.

The PUAP free flap has many advantages, which include its excellent match with fingertips in relation to color, texture, and size. Furthermore, donor site morbidity must be considered. In our patients, the maximum width of the flap was 3.5 cm, the donor sites were closed primarily without any complications, and the scars had healed well after 3 months. Thus, the use of the PUAP free flap does not require the sacrifice of the main vascular pedicle. Omokawa et al. used the ulnar digital artery reverse island flap for fingertip reconstruction, and they pointed out that the sacrifice of the main pedicle can disrupt the arterial supply to the little finger after harvesting a flap with a vascular pedicle [4]. Hwang et al. demonstrated that the mean (±standard deviation) diameter of the PUAP at its origin was 1.55 ± 0.52 mm and that the size matched that of the recipient artery in Zone I fingertip injuries [2]. Gu et al. found venous size mismatches during the reconstruction of fingers with Zone I injuries while performing partial second toe pulp free transfers [1]. However, we did not experience venous size mismatches in the hand, and there were 2-3 subcutaneous veins to cover the flap. Simsek et al. performed toe pulp free transfers for fingertip reconstructions; however, the patients in their study experienced ongoing sensation deficits in their fingers, donor site morbidity, and 2 operation fields [9]. The loss of the tactile sensation in a finger has a fundamental impact on hand function. Hwang et al. demonstrated that there is no sensory nerve on the PUAP, which is one of the major disadvantages of the PUAP flap. However, we found sensory nerve branches covering the flaps in our patients, and the 6-month average 2-point discrimination of 5.7 mm obtained postoperatively from the PUAP free flap reconstruction cases was similar to the results obtained from fingertips repaired using cross-finger flaps (6 mm) and replanted fingers (5.7 mm) [10]. Omokawa et al. reported that the sensory branch in the hand has multiple variations. These anatomic variations and inconsistencies associated with the perforator prevent the use of these two nerves to innervate a sensate flap. The use of the superficial branch of the ulnar nerve should be studied in the future.

Kim et al. divided the hypothenar perforator into 2 parts, namely, the proximal ulnar perforator and the distal ulnar perforator [11]. The proximal ulnar perforator is a true perforator that arises from the superficial palmar arch, that is, the PUAP. However, the distal ulnar perforator may be the perforator that arises from the ulnar palmar digital artery perforator of the little finger. Furthermore, Omokawa et al. reported that the perforator from ulnar palmar digital artery of the little finger was too small and inconsistent [4].

## 5. Conclusions

Fingertip reconstruction using PUAP flaps offers many advantages, including their anatomical consistency as we demonstrated through the cadaveric study. Thus, the authors recommend the use of the PUAP free flap for the reconstruction of fingertips injuries.

## Figures and Tables

**Table 1:** Cadaveric study results from 8 hands.

	Distance from the *x*-axis^*∗*^ (mm)	Diameter at Origin (mm)	Length from the origin (mm)
1	16	0.8	10
2	18	0.9	11
3	20	1.2	9
4	24	0.9	10
5	19	1.0	12
6	18	0.9	13
7	24	0.8	11
8	21	0.7	14
Mean ± SD	20 ± 2.88	0.9 ± 0.15	11.25 ± 1.67

^*∗*^The  *x*-axis is the line between the pisiform and hamate bones.

**Table 2:** Patients' characteristics.

Mean age, years (range)	44 (20–62)
Sex, *n*	
Male	34
Female	10
Injury mechanism, *n*	
Crushing	33
Knife	11
Operation type, *n*	
Primary	37
Secondary	7
Flap size, cm × cm	
Minimum	2.0 × 2.3
Maximum	2.5 × 3.5
Mean operation time, min (range)	124 (98–220)
